# Supplementary material for: Coarse-Grained Modeling of EUV Patterning Process Reflecting Photochemical Reactions and Chain Conformations
Source: Polymers (Basel). 2023 Apr 22;15(9):1988. doi: 10.3390/polym15091988 (PMC10180770; doi:10.3390/polym15091988)
Supplement: Supplementary file 1 [file polymers-15-01988-s001.zip › polymers-2332639-supplementary.pdf]

Supplementary Material

Coarse-Grained Modeling of EUV Patterning Process Reflecting Photochemical Reactions and Chain Conformations

Tae-Yi Kim [1], In-Hwa Kang [3], Juhae Park [1,5], Myungwoong Kim [4,*], Hye-Keun Oh [3,*], Su-Mi Hur [1,2,*]

[1] Department of Polymer Engineering, Graduate School, Chonnam National University, Gwangju 61186, Republic of Korea

[2] School of Polymer Science and Engineering, Chonnam National University, Gwangju 61186, Republic of Korea

[3] Department of Applied Physics, Hanyang University, Ansan 15588, Republic of Korea

[4] Department of Chemistry and Chemical Engineering, Inha University, Incheon 22212, Republic of Korea

[5] Pritzker School of Molecular Engineering, The University of Chicago, Chicago, Illinois 60637, United States

[*] Corresponding author E-mail: shur@chonnam.ac.kr (S.-M.H.); hyekeun@hanyang.ac.kr (H.-K.O.); mkim233@inha.ac.kr (M. K.)

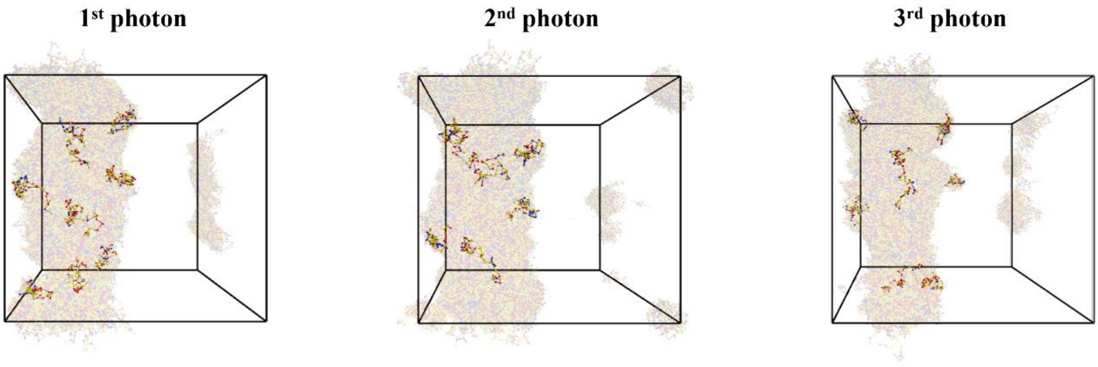

Figure S1. Top views of the first type resist exposed to the three photon distributions after drying the developing solvents. Representative chain conformations at the interface and inside the unexposed resists are presented with unshaded colors.

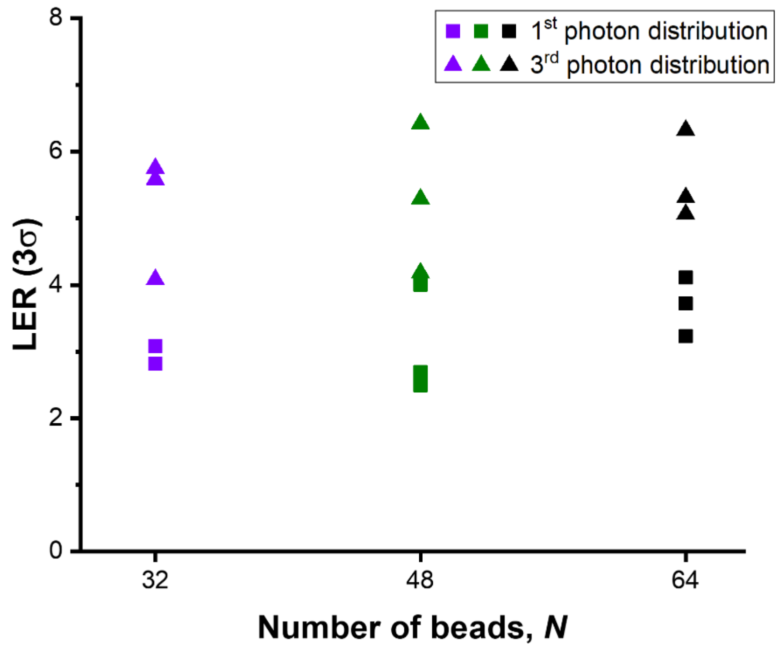

Figure S2. LER as a function of the number of beads, *N* in the photoresists of various random initial material conformations exposed to the 1st and 3rd photon distributions. While the 1st and 3rd photon distributions are represented by the square and triangle, respectively, the resists with three initial material conformations are marked in the same colors.

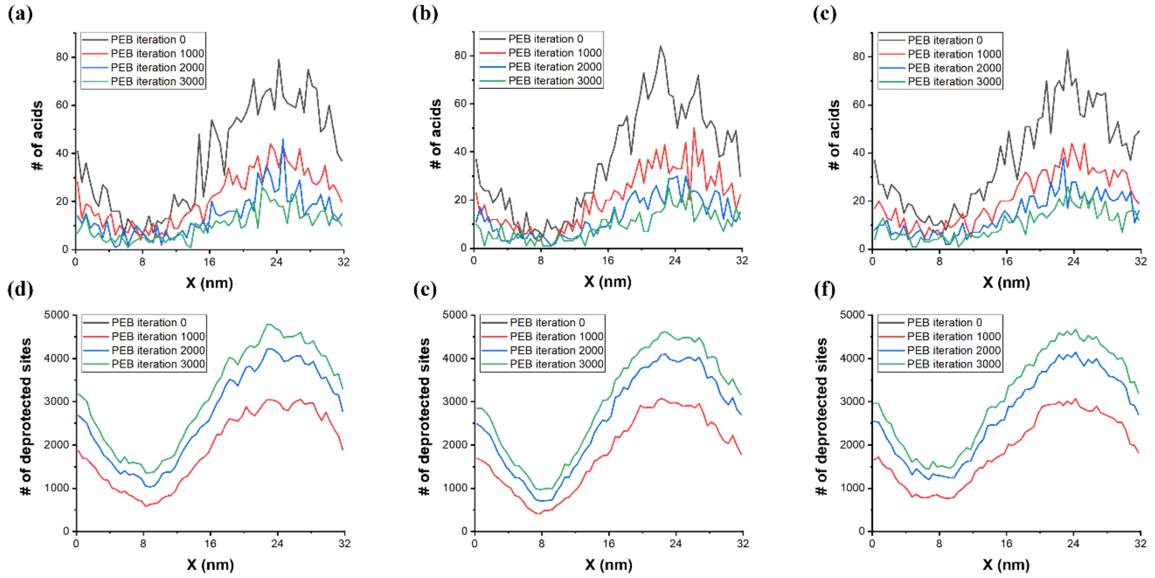

Figure S3. Variation in the distribution of acids (a)-(c) and deprotected sites (d)-(f) in the photoresists exposed to three different photon distributions during the PEB process. The first type of resists is exposed to the 1st, 2nd, and 3rd photon distributions, resulting in the plots of (a) and (d), (b) and (e), (c) and (g), respectively.

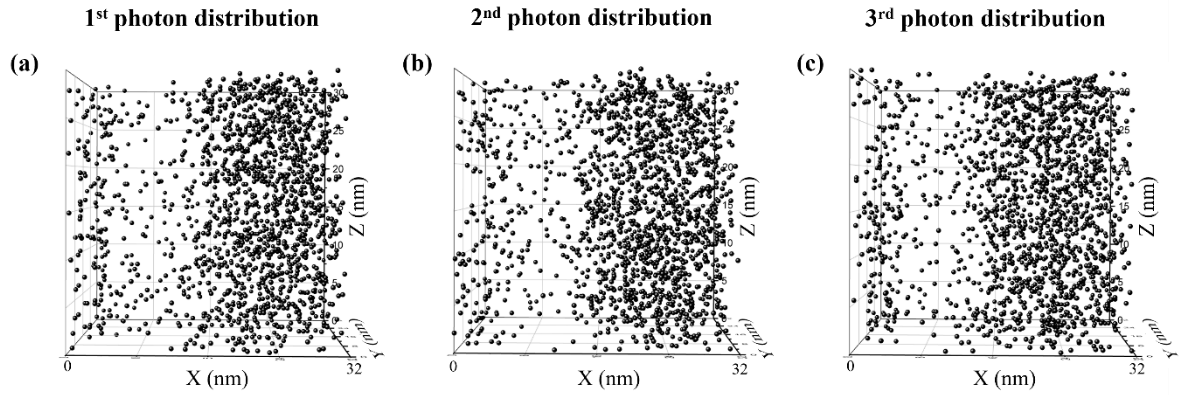

Figure S4. 3D mapping of photon distributions obtained PROLITH which is performed under a Numerical Aperture (NA) of 0.33 and dose of 50 mJ/cm2. The photons are in 32nm × 32nm × 30nm equal to the size of a photoresist.

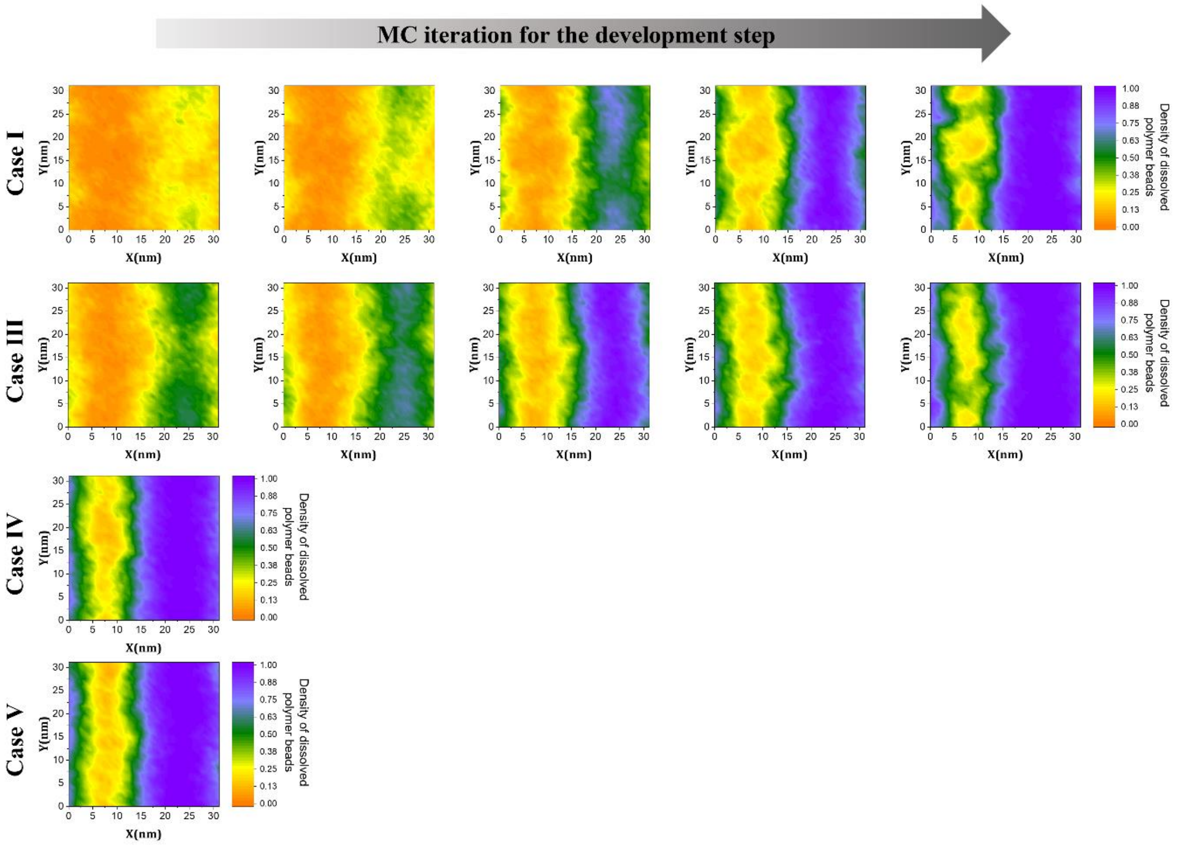

Figure S5. 2D colormaps of the variation in local density of dissolved polymer beads during the development process under the conditions for Case I, III, IV, and V. The densities for the four cases are captured at the same MC iterations of the development and averaged along the same z-direction. Completely dissolved area is represented by the purple.

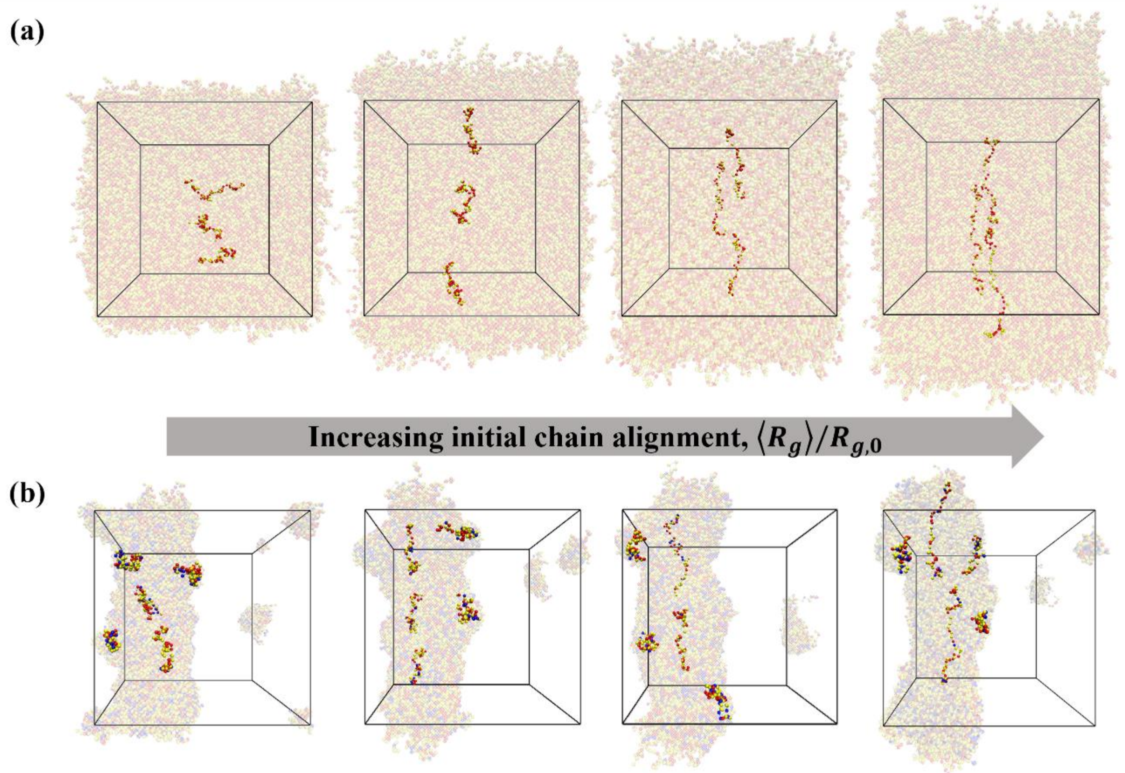

Figure S6. (a) Snapshots of chain conformations in the resists after the chain relaxation of PAB step with increasing $R_g/R_{g,0}$ from 1.0 to 1.47, 1.78, 2.0. (b) The top views of residual resists after drying dissolving solvents are presented with representative chains at the interfaces and inside the resists. The resists are exposed to the 1st photon distribution.

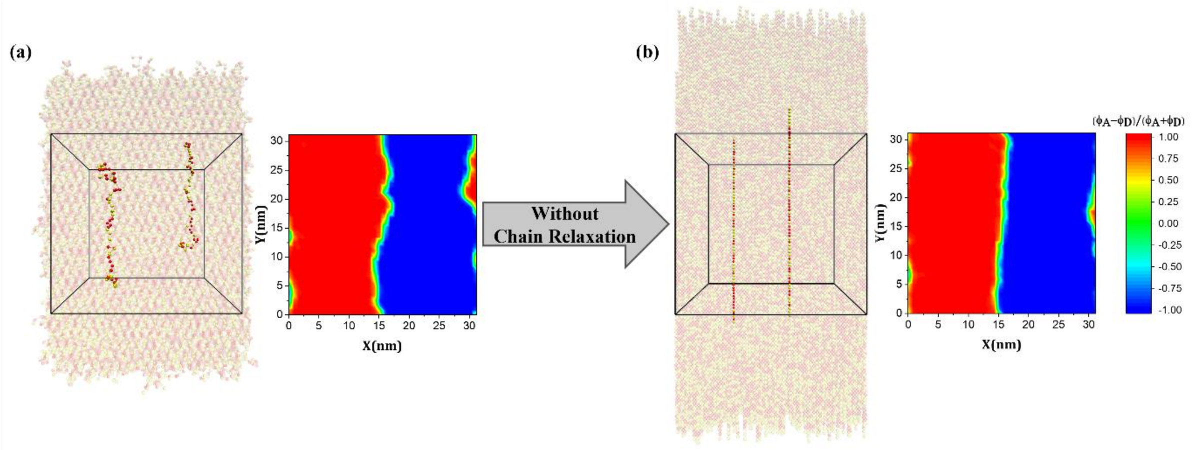

Figure S7. The effect of chain immobility during the PAB step on the final roughness of the photoresist at $R_g/R_{g,0} = 2.0$. (a) The system corresponds to the case of $\langle R_g \rangle / R_{g,0} = 2.0$ with the lowest LER in Figure 6. (b) The resists with representative chain conformations after PAB step are captured. The projected views of residual resists after drying dissolving solvents are presented on the colormap of the normalized local density ($\frac{\boldsymbol{\phi_A - \phi_D}}{\boldsymbol{\phi_A + \phi_D}}$) where the blue and red represent -1 and 1, respectively. The resists are exposed to the 1st photon distribution.

S
